# Ability of fungi isolated from plastic debris floating in the shoreline of a lake to degrade plastics

**DOI:** 10.1371/journal.pone.0202047

**Published:** 2018-08-22

**Authors:** Ivano Brunner, Moira Fischer, Joel Rüthi, Beat Stierli, Beat Frey

**Affiliations:** Forest Soils and Biogeochemistry, Swiss Federal Institute for Forest, Snow and Landscape Research WSL, Birmensdorf, Switzerland; Estacion Experimental del Zaidin, SPAIN

## Abstract

Plastic waste in the environment is a significant threat due to its resistance to biological processes. Here we report the ability of fungal strains found on floating plastic debris to degrade plastics. In particular, we wanted to know which fungi grow on plastic debris floating in the shoreline, whether these fungi have the ability to degrade plastics, whether the plastic-degrading fungi can degrade other complex C-polymers such as lignin, and whether lignin-degraders *vice versa* can also break down plastics. Overall, more than a hundred fungal strains were isolated from plastic debris of the shoreline of Lake Zurich, Switzerland, and grouped morphologically. Representative strains of these groups were then selected and genetically identified, altogether twelve different fungal species and one species of Oomycota. The list of fungi included commonly occurring saprotrophic fungi but also some plant pathogens. These fungal strains were then used to test the ability to degrade polyethylene and polyurethane. The tests showed that none of the strains were able to degrade polyethylene. However, four strains were able to degrade polyurethane, the three litter-saprotrophic fungi *Cladosporium cladosporioides*, *Xepiculopsis graminea*, and *Penicillium griseofulvum* and the plant pathogen *Leptosphaeria sp*. A series of additional fungi with an origin other than from plastic debris were tested as well. Here, only the two litter-saprotrophic fungi *Agaricus bisporus* and *Marasmius oreades* showed the capability to degrade polyurethane. In contrast, wood-saprotrophic fungi and ectomycorrhizal fungi were unable to degrade polyurethane. Overall, it seems that in majority only a few litter-saprotrophic fungi, which possess a wide variety of enzymes, have the ability to degrade polyurethane. None of the fungi tested was able to degrade polyethylene.

## Introduction

Plastic debris in the environment poses a significant threat because of its resistivity to photo-oxidative, thermal, mechanical and biological processes [1,2]. Although overlooked for many years, the amount of plastic debris accumulating in the environment has been steadily increasing as a result of the material's durability and lightweight nature [3,4]. Once discarded on land, plastic debris makes its way to water bodies that act as sinks for low-density litter [5–8]. Topography, wind and water currents, and proximity to pollution sources control the amount and types of plastics along shorelines, whereas degradation processes determine how long plastic debris remains on beaches [9,10].

An estimated 300 million tons of plastic are produced yearly [11]. Plastics are human-made materials manufactured from polymers or long chains of repeating molecules. They are derived from oil, natural gas, and, increasingly, from plants like corn and sugarcane. About four percent of the world’s petroleum is used to make plastic, and another four percent is used to power plastic manufacturing processes [12]. Polyethylene (PE) represent about a third of total plastic production, with PE is largely utilized in packaging [11].

Plastic debris, an inevitable consequence of living the ‘Plastic Age’, is dominating our lakes and oceans and poses a worldwide threat to aquatic wildlife [3,13]. Floating or drifting plastic creates environmental hazards including the risks of plastic ingestion, starvation, and entanglement of aquatic organisms [5,9]. Plastic debris, as recently published from the Lake Geneva, consisted of various size and colour, including bottles, bottle tops, cotton buds, pens, toys, straws, and pieces or blocks of expanded polystyrene or polyurethane foam [14]. Plastic debris also provides novel aquatic vehicles for a wide range of rafting species, such as bacteria, fungi, algae, or insects, posing a potential threat to introduce invasive species [13]. Once plastics are discharged into aquatic environments, they can persist for up to 50 years, and their complete mineralisation may take hundreds or thousands of years [15].

In 2011 US researchers discovered an endophytic fungal species, which was able to degrade polyurethane (PU), a plastic which is widely used in the manufacture of e.g. high-resilience foam seating, rigid foam insulation panels, or tires such as skateboard wheels [16]. This discovery obtained a high attention in the media (e.g. http://www.dailymail.co.uk/sciencetech/article-2146224/Could-fungi-break-plastic-stop-modern-scourge.html). The question arises, what about the fungi which can be found on plastic debris? We had the hypothesis that at least some fungi that grow on plastic debris have the potential to degrade plastics, and that the fungi that can degrade plastics are more generalists than specialists. In particular, we wanted to answer the following questions: (1) Which fungi grow on plastic debris floating in the shoreline? (2) Do the fungi isolated from floating plastics have the ability to degrade PE or PU? (3) Do fungi which are able to degrade plastics also have the ability to degrade other complex C-polymers such as lignin? (4) Do fungal lignin-degraders *vice versa* have the capability to degrade PE or PU? With this study we also had the intention to clarify the abilities of the various trophic modes of fungi (saprotrophs, pathotrophs, symbiotrophs) with its functional guilds (e.g. plant pathogens, wood saprotrophs, [17]) for the degradation of PE or PU.

## Materials and methods

### Sampling of plastic debris

Plastic debris was collected in the shoreline of Lake Zurich close to Wädenswil (UTM coordinates 32T 474250 5231960) at September 2^nd^ 2015. The plastic pieces either floated on the water or were found in a depth of up to 20 cm in the reed belt. The pieces of plastic were picked up with a pair of tweezers and a 0.7 cm x 0.7 cm piece was cut out with a pair of scissor. That piece was then placed into a sterile 50 ml Falcon tube. Before use, tweezers and scissors were dipped into 70% ethanol and flamed over a lighter for sterilization. The falcon tubes were kept sealed in a refrigerated bag and transported the same day to the lab where they were kept at 4°C until use. In total, 13 pieces of plastics were sampled out of the water of the lake, and one was found close to the water (No. 16, Table 1). One piece was a hard-plastic chunk and only a 2.5 cm^2^ fragment could be turned off with the tweezers.

**Table 1:** List of plastic debris collected from the lake of Zurich and the suspected plastic types polyethylene (PE) and polypropylene (PP) (according to Gosh et al. [57]).

No.	Suspected origin of the plastic debris	Suspected plastic type
1	White plastic bag	PE
2	White plastic bag	PE
3	White drinking plastic beaker	PP
4	Transparent/blue plastic packaging for beer cans	PE
5	Transparent plastic packaging	PE
6	Solid blue plastic fragment	PP
7	Transparent plastic packaging	PE
8	Transparent/green plastic packaging for a chewing gum	PE
9	White plastic packaging for ice-cream	PE
10	Transparent re-sealable zipper storage bag	PE
11	Yellow coloured plastic packaging for biscuits	PE
15	White/black plastic packaging for a chocolate bar	PE
16	Solid white plastic fragment	PP

### Isolation of fungi

In the laboratory, 2 ml of sterile water was added to each Falcon tube containing one plastic debris piece. Then the tubes were mixed using a vortex mixer for about 10 s to allow the fungal hyphae and spores to separate from the plastic samples. In the sterile bench, 100 μl of water from each Falcon tube was taken with a sterile pipette, released into a Petri dish containing modified Melin-Norkrans (MMN) nutrient agar, and spread with a flamed glass rod on the surface of the agar (compare also [18]). Per Falcon tube, four Petri dishes were incubated. The plates were then incubated at room temperature in the dark until after a few days the first fungal colonies were visible. Emerging fungal colonies were then punched out with a flamed hook, transferred onto a malt agar in glass tubes ('test-tubes') and incubated at room temperature in the dark.

### Identification of fungi

Once the fungal mycelia in the glass tubes covered about half of the agar surface, they were transferred to 4°C to stop growth. In order to select fungi for DNA identification, the isolated fungal strains were morphologically grouped according to their external appearance in terms of colour and texture. Representative strains of these groups were selected, and a part of the nuclear small subunit rDNA was sequenced. Samples of the fungal mycelia were directly placed into the wells of 96-well PCR-plates containing 100 μl DNAse/RNAse free PCR-grade water per well. Then the fungal hyphae were frozen by submerging the plates into liquid N_2_ and thawed at room temperature at least three times in order to break up the cells and to release the DNA. This solution was then 1:10 diluted in PCR-grade water and used as template for the PCR reaction, performed with the G2 Hot Start Polymerase (Promega AG, Dübendorf, Switzerland), MgCl_2_, dNTP, BSA and the primer pair ITS3/ITS4 [19] similar as in [20]. The resulting PCR products were then sequenced by a company (GATC Biotech, Köln, Germany), and the obtained nucleotide sequences blasted using the National Centre of Biotechnology Information (NCBI) database to obtain the closest species match.

In order to obtain longer DNA fragments for a more precise identification, the fungi, which were able to degrade PE or PU, were sequenced again (Macrogen Europe Amsterdam, the Netherlands) with the primer pair ITS1/ITS4 [19]. These nucleotide sequences were deposited at the NCBI GenBank.

### Degradation assays

The ability of the fungi to degrade plastics was tested with degradation assays in Petri dishes on agar medium. The degradation assay using polyethylene (PE) as a plastic source was done according to Yamada-Onodera et al. [21]. The agar medium contained 3 g L^-1^ NH_4_NO_3_, 5 g L^-1^ K_2_HPO_4_, 1 g L^-1^ NaCl, 0.2 g L^-1^ MgSO_4_.7H_2_O, 0.25 ml L^-1^ Tweed 20, and 15 g L^-1^ agar. Thus, the medium contained the nutrients nitrogen, phosphorus, sulphur, potassium, magnesium, sodium, and chlorine. Immediately after autoclaving, 10 g L^-1^ PE powder (Sigma-Aldrich, Buchs, Switzerland; particle size 125 μm), which was prior to use additionally ground with a mortar in liquid N_2_, was added. The degradation assay using polyurethane (PU) as a plastic source was done according to Russel et al. [16] and Biffinger et al. [22] with the addition of nutrients according to Yamada-Onodera et al. [21]. The agar medium contained 3 g L^-1^ NH_4_NO_3_, 5 g L^-1^ K_2_HPO_4_, 1 g L^-1^ NaCl, 0.2 g L^-1^ MgSO_4_^.^7H_2_O, and 15 g L^-1^ agar. Immediately after autoclaving, 10 ml l^-1^ PU was added. The PU used was Impranil^®^DLN-SD, Bayer MaterialScience (CSC JÄKLECHEMIE GmbH & Co. KG, Nürnberg, Germany), which is a polyester polyurethane dispersion.

The ability of the fungi to degrade a complex C-polymer other than plastic, e.g. lignin, was tested with the 'Bavendamm' assay in Petri dishes on agar medium [23]. This assay uses polyphenols as a lignin substitution. The agar medium contained 20 g L^-1^ malt extract and 15 g L^-1^ agar, and as a polyphenol, 0.5 g L^-1^ tannic acid (TA) was added to the solution before autoclaving [23].

**Fig 1:**
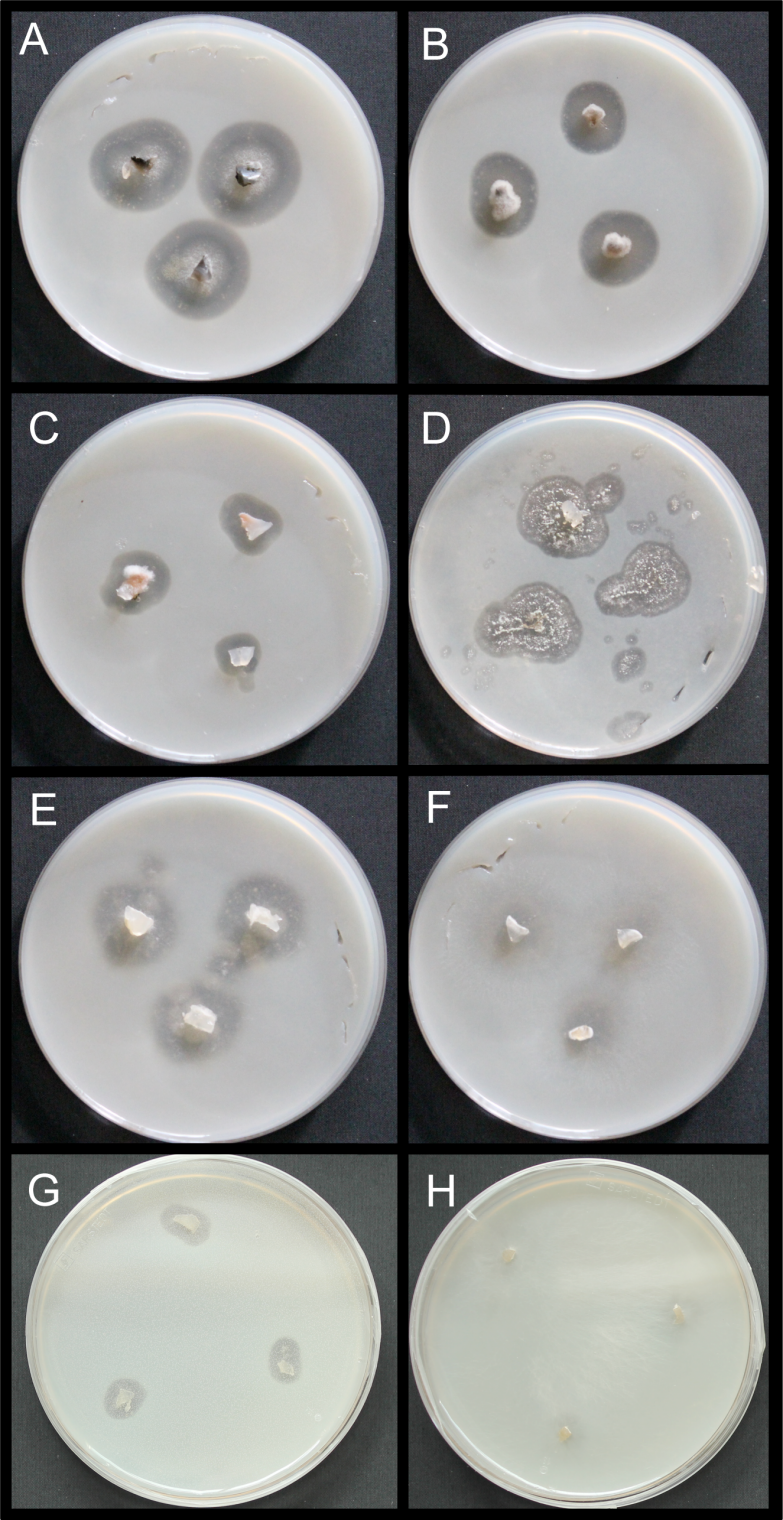
Degradation of polyurethane (PU) in Petri dishes (diameter 9 cm) by fungal inoculi at room temperature. (A) Degradation of PU (halo) after 6 days of growth by the fungus *Cladosporium cladosporioides* (WSL No. 156.01). (B) Degradation of PU (halo) after 6 days of growth by the fungus *Leptosphaeria sp*. (WSL No. 165.01). (C) Degradation of PU (halo) after 6 days of growth by the fungus *Xepiculopsis graminea* (WSL No. 155.01). (D) Degradation of PU (halo) after 6 days of growth by the fungus *Penicillium griseofulvum* (WSL No. 159.01). (E) Degradation of PU (halo) after 6 days of growth by the fungus *Pestalotiopsis microspora* (WSL No. 147.01). (F) Degradation of PU (halo) after 6 days of growth by the fungus *Marasmius oreades* (WSL No. 105.01). (G) Degradation of PU (halo) after 14 days of growth by the fungus *Agaricus bisporus* (WSL No. 99.01). (H) No degradation of PU after 14 days of growth by the white-rot fungus *Pleurotus eryngii* (WSL No. 130.01).

Into each of the Petri dishes, three inoculi per fungal strain were placed on the media (compare also Fig 1). Then, the dishes were sealed with plastic paraffin film and incubated at room temperature in the dark. The Petri dishes were visually inspected every few days.

### Optical evaluation of the degradation

The media in Petri dishes containing PE or PU were both milky and not transparent. The PE polymers, however, floated on the top of the medium during agar solidification, whereas PU polymers remained homogeneously distributed within the medium after agar solidification. According to Russell et al. [16] it was expected that fungi capable of degrading the plastic polymers would display a zone of clearance ('halo') around the growing cultures as a result of enzymatic plastic degradation by diffusing enzymes excreted by the fungal hyphae, or in the case of PE, grow on the plastic granules [21]. The media with the TA, however, was expected to change the colour from light brown to dark brown as a result of an enzymatic oxidative reaction of the TA by diffusing enzymes excreted by the fungal hyphae [23].

### Fungus species from the fungal collection for degradation assays

Twenty-one fungal species of the WSL (Swiss Federal Institute for Forest, Snow and Landscape Research) fungal collection belonging to different ecological guilds [17] were selected and tested for its PE, PU, and TA degradation ability, e.g. common saprotrophs (e.g. *Agaricus bisporus*), wood saprotrophs ('white rots': e.g. *Phanerochaete sanguinea*, *'*brown rots': e.g. *Fomitopsis pinicola*), tree pathogens (e.g. *Heterobasidion parviporum*), and ectomycorrhizal fungi (e.g. *Suillus granulatus*). The distinction of wood-decomposing fungi between 'white rot' and 'brown rot' fungi followed Breitenbach and Kränzlin [24] and Gramss et al. [23], with 'white rot' fungi being able to degrade lignin, but the 'brown rot' fungi not.

In order to have a control strain for the degradation of PU, *Pestalotiopsis microspora* was purchased from the Westerdijk Fungal Biodiversity Institute (CBS No. 364.54; CBS-KNAW, Utrecht, The Netherlands). *P*. *microspora* is able to degrade PU [16].

## Results

### Fungal strains isolated from plastic debris

Fungal strains usually grew within a few days after dispersing the water from the Falcon tubes on the Petri dishes. In total, more than one hundred fungal strains were isolated. According to their external appearance, the fungal strains were grouped into morphological groups. From these groups, one or two fungal strains per group were selected, in total 24 fungal strains, and a part of the nuclear small subunit rDNA was sequenced. After blasting the sequences with the NCBI database, the names of the closest species match were listed. In several cases, identical names appeared. The final list of organisms isolated and sequenced from plastic debris contained twelve different fungal species belonging to the Ascomycota and one species to the Oomycota (*Pythium)* (Table 2). The fungal names were checked and approved using the 'Index Fungorum' (http://www.indexfungorum.org). A good identification of fungal names is given when the nucleotide identity was equal or above 97% [25]. If the identity was below 97%, then the names have to be taken with caution, and they might not be correct (Table 2).

**Table 2:** List of fungi isolated from plastic debris and their ability to degrade polyethylene (PE) and/or polyurethane (PU), respectively (+ yes;—no). The fungi were sequenced with the primer pairs ITS1/ITS4 or ITS3/ITS4. Living cultures are deposited at the WSL fungus collection. WSL No.: Number of the culture in the WSL fungus collection, Length: Length of the sequenced fragment (bp = base pairs), (%) with the closest match of the NCBI database having a genus name.

Species identity	Fungus species	WSL No.	Primer	Length (bp)	Identity (%)	Closest NCBI	PE	PU
High	*Arthrinium arundinis*	167.01	ITS3/4	301	99	KJ188680.1	-	-
High	*Botryotinia fuckeliana*^a^	168.01	ITS3/4	300	99	KF533003.1	-	-
High	*Cladosporium cladosporioides*^b^	156.01	ITS1/4	522	99	KU508795.1	-	+
High	*Leptosphaeria sp*.^c^	165.01	ITS1/4	495	99	KP747710.1	-	+
High	*Penicillium griseofulvum*	159.01	ITS3/4	314	99	KJ467353.1	-	+
High	*Phialemoniopsis curvata*^d^	166.01	ITS3/4	306	98	NR132067.1	-	-
High	*Phoma sp*.	163.01	ITS3/4	306	99	DQ344033.1	-	-
High	*Pythium phragmitis*	162.01	ITS3/4	584	98	HQ643746.1	-	-
High	*Stagonospora neglecta*	169.01	ITS3/4	303	99	AJ496630.1	-	-
High	*Xepiculopsis graminea*^e^	155.01	ITS1/4	546	97	HQ608010.1	-	+
Low	*Exophiala bonariae*	160.01	ITS3/4	347	96	KP791795.1	-	-
Low	*Pseudorobillarda texana*	164.01	ITS3/4	326	84	FJ825372.1	-	-
Low	*Setophoma vernoniae*	158.01	ITS3/4	309	88	KJ869141.1	-	-

^a^Synonym: *Botrytis fuckeliana*

^b^NCBI accession No.: MF327241.1

^c^NCBI accession No.: MF327242.1

^d^Same identities for *Tricladium patulum*, FJ000403.1; *Sarocladium strictum*, AY138486.1; *Acremonium sp*., HM535388.1

^e^NCBI accession No.: MF327243.1; Synonym: *Myrothecium gramineum*

### Ability of the fungal strains from plastic debris to degrade PE and PU

In total, twelve fungus species and one species of Oomycota were tested to degrade PE or PU (Table 2). After at least three weeks of growth, neither signs of 'halos' were visible around the inoculi nor growth of the inoculi was recorded in the PE degradation assay. I contrast, 'halos' were visible in the PU degradation assay after at least three weeks of growth around the inoculi of four fungal species *Cladosporium cladosporioides*, *Xepiculopsis graminea*, *Penicillium griseofulvum*, *and Leptosphaeria sp*. (Table 2, Fig 1). The most efficient fungi for PU degradation was *C*. *cladosporioides* with an approximate growth of the halo of 4 mm/d (Fig 1). To ensure the species names, some of these taxa were sequenced again with the primer pairs ITS1 and ITS4 to obtain longer sequences, which then were deposited at the NCBI database under the accession numbers MF327241—MF327243 (see also Table 2).

### Ability of fungal strains from various fungal guilds to degrade PU and TA

Overall, none of the tested fungi was able to degrade PE (data not shown). However, three fungal species were able to degrade PU: *Agaricus bisporus*, *Marasmius oreades*, and *Pestalotiopsis microspora* (Table 3). Surprisingly, none of the highly specialised lignin-decomposing fungi such as the saprotrophic white-rot fungi or the plant pathogens were able to degrade PU. Similarly, the ectomycorrhizal fungi as well as the saprotrophic brown-rot fungi were not able to degrade PU. From the common saprotrophs, who all were able to degrade TA, only the two species *A*. *bisporus* and *M*. *oreades* were able to degrade additionally PU (Table 3).

**Table 3:** Selected fungi of the WSL (Swiss Federal Institute for Forest, Snow and Landscape Research) culture collection isolated from plastic debris or from various other sources and their ability to degrade polyurethane (PU) and/or tannic acid (TA), respectively (+ yes;—no). Guild: Ecological groups according to Nguyen et al. [17] and Gramss et al. [23]. Wood decomposers: BR: Brown rot fungi, WR: White rot fungi, WSL No.: Number of the culture in the WSL fungus collection.

Origin of isolation	Phylum	Guild	Fungus species	WSL No.	PU	TA
Plastic debris	Ascomycota	Litter-saprotroph	*Cladosporium cladosporioides*	156.01	+	-
Plastic debris	Ascomycota	Litter saprotroph	*Xepiculopsis graminea*	155.01	+	+
Plastic debris	Ascomycota	Litter-saprotroph	*Penicillium griseofulvum*	159.01	+	-
Plastic debris	Ascomycota	Plant pathogen	*Leptosphaeria sp*.	165.01	+	-
Plastic debris	Ascomycota	Plant pathogen	*Arthrinium arundinis*	167.01	-	-
Plastic debris	Ascomycota	Plant pathogen	*Botryotinia fuckeliana*	168.01	-	-
Plastic debris	Ascomycota	Endophyte	*Stagonospora neglecta*	169.01	-	+
Plant substrate	Ascomycota	Endophyte	*Pestalotiopsis microspora*^a^	147.01	+	-
Fruiting body	Basidiomycota	Litter-saprotroph	*Agaricus bisporus*	99.01	+	+
Fruiting body	Basidiomycota	Litter-saprotroph	*Marasmius oreades*	105.01	*+*	*+*
Fruiting body	Basidiomycota	Litter-saprotroph	*Agrocybe praecox*	125.01	-	+
Fruiting body	Basidiomycota	Litter-saprotroph	*Clitocybe nebularis*	103.01	-	+
Fruiting body	Basidiomycota	Litter-saprotroph	*Coprinus comatus*	149.01	-	+
Fruiting body	Basidiomycota	Litter-saprotroph	*Phallus impudicus*	128.01	-	+
Fruiting body	Basidiomycota	Wood-saprotroph-WR	*Hypholoma fasciculare*	153.01	-	+
Fruiting body	Basidiomycota	Wood-saprotroph-WR	*Armillaria cepistipes*	129.01	-	+
Fruiting body	Basidiomycota	Wood-saprotroph-WR	*Phanerochaete sanguinea*	140.01	-	+
Fruiting body	Basidiomycota	Wood-saprotroph-WR	*Pleurotus eryngii*	130.01	-	+
Fruiting body	Basidiomycota	Wood-saprotroph-WR	*Pleurotus ostreatus*	134.01	-	+
Fruiting body	Basidiomycota	Wood-saprotroph-WR	*Stereum hirsutum*	136.01	-	+
Fruiting body	Basidiomycota	Plant pathogen-WR	*Armillaria ostoyae*	135.01	-	+
Fruiting body	Basidiomycota	Plant pathogen-WR	*Climacocystis borealis*	132.01	-	+
Fruiting body	Basidiomycota	Plant pathogen-WR	*Heterobasidion parviporum*	131.01	-	+
Fruiting body	Basidiomycota	Wood-saprotroph-BR	*Fomitopsis pinicola*	142.03	-	-
Fruiting body	Basidiomycota	Wood-saprotroph-BR	*Gloeophyllum sepiarium*	80.01	-	-
Fruiting body	Basidiomycota	Wood-saprotroph-BR	*Postia tephroleuca*	141.01	-	-
Fruiting body	Basidiomycota	Ectomycorrhizal	*Hebeloma edurum*	8.01	-	-
Fruiting body	Basidiomycota	Ectomycorrhizal	*Suillus granulatus*	144.01	-	-

^a^CBS No. 364.54

Of the four fungal species isolated from the plastic debris and able to degrade PU, *Xepiculopsis graminea* was the only species that was able to degrade TA. The endophytic *P*. *microspora*, our PU-degradation reference strain [16], was not able to degrade TA (Table 3).

Besides the PU-degrading fungi reported in the present study, 15 ascomycete fungi are reported to potentially degrade PU (Table 4). The best-known fungi are members of the genera *Aspergillus*, *Penicillium*, and *Trichoderma*. Two ascomycete and two basidiomycete fungi from this study are newly reported to be able to degrade PU.

**Table 4:** List of fungal species able to degrade polyurethane (PU).

Phylum	Fungus species	Reference
Ascomycota	*Alternaria alternata*	[61]
Ascomycota	*Aspergillus fumigatus*, *A*. *niger*	[61]
Ascomycota	*Aureobasidium pullulans*	[1]
Ascomycota	*Cladosporium cladosporioides*	[58], this study
Ascomycota	*Colletotrichum gloeosporioides*	[61]
Ascomycota	*Corynespora cassiicola*	[61]
Ascomycota	*Curvularia senegalensis*	[1]
Ascomycota	*Fusarium moniliformae*, *F*. *solani*	[61]
Ascomycota	*Geomyces pannorum*	[39]
Ascomycota	*Lasiodiplodia crassispora*, *L*. *theobromae*	[61]
Ascomycota	*Leptosphaeria sp*.	This study
Ascomycota	*Nectria gliocladioides*	[39]
Ascomycota	*Penicillium ochrochloron*, *P*. *griseofulvum*	[39], this study
Ascomycota	*Periconia sp*.	[61]
Ascomycota	*Pestalotiopsis microspora*	[16]
Ascomycota	*Trichoderma harzianum*	[61]
Ascomycota	*Xepiculopsis graminea*	This study
Basidiomycota	*Agaricus bisporus*	This study
Basidiomycota	*Marasmius oreades*	This study

## Discussion

The list of organisms, which have been isolated from plastic debris, included commonly occurring saprotrophic fungi but also some plant pathogens. Commonly occurring saprotrophic fungi were *Penicillium griseofulvum* and *Cladosporium cladosporioides* [26,27]. Some fungi are known to live as saprotrophs in soils and sediments such as *Xepiculopsis graminea* and *Phialemoniopsis curvata* [28,29]. Some fungi are known to live in association with grasses or with plants growing in the littoral zones of lakes, e.g. *Arthrinium arundinis*, *Leptosphaeria sp*. and *Phoma sp*. [30,31]. Some fungal species are highly specialised to the common reed (*Phragmites australis)* such as the endophytic fungus *Stagonospora neglecta* [32]. *Botryotinia fuckelinana* is known as a necrotrophic fungus that affects many plant species [33]. The fungal species, which only had a low identity (*Exophiala bonariae*, *Pseudorobillarda texana*, *Setophoma vernoniae*), were isolated by others either from rocks or from leaves of exotic plants [28,34]. The only organism not belonging to the fungi was the oomyceteous *Pythium phragmitis* which is a pathogen for the common reed (*Phragmites australis)* [35].

The four fungal species isolated from plastic debris showed a 'halo' in the PU assay: *C*. *cladosporioides*, *P*. *griseofulvum*, *X*. *graminea*, *and Leptosphaeria sp*. *Cladosporium cladosporioides* had been observed already by others to be able to degrade PU. Álvarez-Barragán et al. [36] found that the six best PU-degrading strains using an Impranil assay belonged to the *C*. *cladosporioides* complex, with identities between 99% and 100%. Further BLAST analysis of the actin and translation elongation factor from these six strains showed the highest matches with the *C*. *pseudocladosporioides*, *C*. *tenuissimum*, *C*. *asperulatum*, and *C*. *montecillanum* [36]. Some reports, in contrast to our study, stated that *C*. *cladosporioides* is able to degrade PE as well (e.g. [37,38]). However, their results based not on the formation of a 'halo' in a Petri dish after PE degradation, but on observing erosion of the PE film surface in the vicinity of the fungal hyphae as well as formation of oxidation products in the surface of the polymer film measured by FTIR (Fourier-transform infrared spectroscopy). *Penicillium ochrochloron*, a different species than our isolated *P*. *griseofulvum*, had been observed already by other authors to have the capability to degrade PU [39]. These authors applied similarly as described above the PU assay using Impranil for soil fungi which they isolated from soil-buried PU pieces. For *Xepiculopsis graminea* and *Leptosphaeria sp*., in contrast, no references were found in the literature. Thus, this is the first report on the ability of these two fungi to degrade PU.

There is evidence from the literature that microorganisms capable of degrading complex C polymers such as lignin can also degrade plastics [40]. Such degradation potential is based on lignin-degrading enzymes, e.g. oxidases, laccases and peroxidases, which are used in various industries and which are also reported to be involved in the degradation of xenobiotic compounds and dyes [41]. Overall, the three saprotrophic fungi *Agaricus bisporus*, *Marasmius oreades* and *Xepiculopsis graminea* remain the only fungi in our study which were able to degrade PU as well as TA. At least *A*. *bisporus* is known to possess a wide variety of enzymes including enzymes involved in xylan, cellulose, pectin, and protein degradation, as well as heme-thiolate peroxidases and β-etherases, which are distinctive from other wood-decayers and suggest a broad attack on decaying lignin and related metabolites found in humic acid-rich environment [42]. The catabolic ability of *A*. *bisporus* agrees with the presence of a large set of genes encoding CAZymes [43] acting on cell wall polysaccharides including glycoside hydrolases, polysaccharide lyases, and carbohydrate esterases [42]. Carbohydrate esterases are suited in *A*. *bisporus* to break down the cell wall polysaccharides xylan, chitin, and pectin [44].

*Marasmius oreades* is known to produce fairy rings in grasslands. Fairy rings are characterised by two or three adjacent concentric zones of abnormal turf. Within the zone of most intense fungal growth, the grass is often killed, and this effect has been attributed to a lack of moisture and to hydrocyanic acid produced by the fungus [45,46]. The occurrence of fairy rings in natural vegetation has simultaneous contrasting effects of both stimulation and a parasitisation of plant species in adjacent zones, producing concentric regular bands of lush and scorched vegetation [47]. In soils colonized by *M*. *oreades*, degradation of plant roots in the presence of fungal cell-wall degrading enzymes increased the content of dissolved organic carbon [48]. Interestingly, similar as *M*. *oreades*, members of the *Agaricus* genus form fairy ring as well, e.g. *A*. *arvensis* [49]. Thus, it can be assumed, that members of both, *Agaricus* and *Marasmius*, possess similar enzymatic capabilities to break down complex carbohydrate polymers.

Members of *Xepiculopsis* are filamentous ascomycete fungi, which grow ubiquitous in soils or are weak plant pathogens, but they also are capable of growing on walls in houses [50]. Some species produce mycotoxins and are used as bio-control agents to control weeds [51,52]. *Xepiculopsis graminea* was originally described as *Myrothecium gramineum* on decaying grasses [53]. But other than that, not much is known from this species.

Besides the PU-degrading fungi reported in the present study, a series of other ascomycete fungi are reported to degrade PU (Table 4). The best-known fungi are members of the genera *Aspergillus* and *Trichoderma*, all of which are known to be used in biotechnological processes [54]. Members of *Aspergillus* are used to produce the enzymes amylases, glucoamylases, glucose oxidase, invertase, pectinase, and proteinases, whereas members of *Trichoderma* are used to produce cellulase [55].

Although we have in the present study not investigated ourselves the enzymes produced by the PU-degrading fungi, there are several studies which report that enzymes involved in PU degradation are most likely esterases and hydrolases. Alvarez-Barragan et al. [36] postulated that *Cladosporium cladosporioides* complex were the best PU degraders among the fungi tested, whereas *Aspergillus fumigatus* and *Penicillium chrysogenum* were the least degrading strains. Besides Impranil, the fungal isolates of *Cladosporium spp*. degraded PU foam as well. FTIR spectroscopy and GC-MS analysis showed that ester and urethane groups were attacked through the activity of fungal enzymes. During PU degradation, considerable activities of esterases were detected, but only low urease and no protease activities [36]. Loredo-Treviño et al. [56], isolating 32 fungal strains from sand samples contaminated with PU, reported 22 strains being able to grow using PU as nutrient source. Among the genera found were *Aspergillus*, *Trichoderma*, *Penicillium*, and *Fusarium*. Almost all of the PU-degrading fungi showed urease activity, whereas esterase, protease, and laccase activities were present only in a lower amount of the fungi. For the PU-degrader species *Pestalotiopsis microspora*, Russell et al. [16] suggested a serine hydrolase-like enzyme being responsible for PU degradation.

## Conclusions

The majority of fungi isolated from plastic debris in the shoreline of a lake in Switzerland do not seem to be able to degrade the plastic they grew on. None of the fungi was able to degrade PE, whereas at least a few fungi isolated had the ability to degrade PU. Three of these fungi were saprotrophs, and one was a plant pathogen. Thus, we could only partially confirm the previously formulated hypothesis that at least some of the fungus can degrade plastic, but only PU and not especially PE. The search for additional fungal species isolated from other substrates than from plastics or from fruiting bodies revealed that they were in general as well unable to degrade PE. Only two saprotrophic fungi, *Agaricus bisporus* and *Marasmius oreades*, were able to degrade PU.

It seems that the biological degradation of PE still remains a challenge. Although there have recently been several review articles that fungi can degrade PE (e.g. [1,40,57–62]), these reports are no more than vague hints. Otherwise, the plastic waste would not be transported vertically across oceans and landscapes, mechanically fragmented, and eventually accumulated as micro- or nanoplastic in the sediments and environment if fungi and other microorganisms were efficient in degradation. Although the majority of plastic debris that has entered the ocean since 1950 has settled to depths below the ocean surface layer, it is estimated that 0.3 million tons of plastic are floating on the ocean surface, of which an estimated 14% is microplastic (0.335–5 mm) and 2.5% is nanoplastic (<0.335 mm) [63]. These small plastic fragments in particular are problematic, because they enter into the food webs and accumulate potentially in animals [64–68].

Knowing the ecological guild would facilitate the search for potential fungi which are able to degrade plastics. Not long ago a Japanese group found in a similar way a bacterium that degrades poly(ethylene terephthalate) PET [69]. If such microorganisms could be found, their spores or their plastic degrading enzymes could be incorporated into the plastic material during manufacturing and, when the plastic waste would come into contact with lake- or sea-water, the fungi would start to grow and to degrade the plastic.
